# Sex difference and outcome trends following transcatheter aortic valve replacement

**DOI:** 10.3389/fcvm.2022.1013739

**Published:** 2022-10-18

**Authors:** Gabby Elbaz-Greener, Eldad Rahamim, Zahi Abu Ghosh, Shemy Carasso, Merav Yarkoni, Sam Radhakrishnan, Harindra C. Wijeysundera, Tomas Igor, David Planer, Guy Rozen, Offer Amir

**Affiliations:** ^1^Department of Cardiology, Hadassah Medical Center, The Faculty of Medicine, Hebrew University of Jerusalem, Jerusalem, Israel; ^2^Division of Cardiovascular Medicine, Baruch Padeh Medical Center, Poria, Israel; ^3^The Azrieli Faculty of Medicine in the Galilee, Bar-Ilan University, Safed, Israel; ^4^Schulich Heart Program, Division of Cardiology, Sunnybrook Health Sciences Centre, University of Toronto, Toronto, ON, Canada; ^5^Institute for Cardiovascular Disease of Vojvodina, Sremska Kamenica Institute, Belgrade, Serbia; ^6^Cardiovascular Center, Tufts Medical Center, Boston, MA, United States; ^7^Tufts University School of Medicine, Boston, MA, United States; ^8^Cardiac Arrhythmia Center, Harvard Medical School, Massachusetts General Hospital, Boston, MA, United States

**Keywords:** TAVR, aortic valve replacement, transcatheter aortic valve replacement, gender, interventional cardiology

## Abstract

**Background:**

Based on worldwide registries, approximately 50% of patients who underwent transcatheter aortic valve replacement (TAVR) are female patients. Although TAVR procedures have improved tremendously in recent years, differences in outcome including mortality between sexes remain. We aimed to investigate the trends in TAVR in the early and new eras of utilization and to assess TAVR outcomes in female patients vs. male patients.

**Methods:**

Using the 2011–2017 National Inpatient Sample (NIS) database, we identified hospitalizations for patients with the diagnosis of aortic stenosis during which a TAVR was performed. Patients' sociodemographic and clinical characteristics, procedure complications, and mortality were analyzed. Piecewise regression analyses were performed to assess temporal trends in TAVR utilization in female patients and in male patients. Multivariable analysis was performed to identify predictors of in-hospital mortality.

**Results:**

A total of 150,647 hospitalizations for TAVR across the United States were analyzed during 2011–2017. During the study period, a steady upward trend was observed for TAVR procedures in both sexes. From 2011 to 2017, there were significantly more TAVR procedures performed in men [80,477 (53.4%)] than in women [70,170 (46.6%)]. Male patients had significantly higher Deyo-CCI score and comorbidities. Differences in mortality rates among sexes were observed, presenting with higher in-hospital mortality in women than in men, OR 1.26 [95% CI 1.18–1.35], *p* < 0.001.

**Conclusion:**

Utilization of TAVR demonstrated a steady upward trend during 2011–2017, and a similar trend was presented for both sexes. Higher in-hospital mortality was recorded in female patients compared to male patients. Complication rates decreased over the years but without effect on mortality differences between the sex groups.

## Introduction

Aortic stenosis (AS) is characterized by left ventricular outflow obstruction. This results in decreased cardiac output, which leads to major morbidity and mortality. AS is a progressive disease. The prevalence in octogenarians is 9.8 vs. 0.2% in adults aged 50–59 years, which suggests degenerative etiology as the main cause of the disease (1). Aortic valve replacements (AVR) *via* surgical aortic valve replacements (SAVR) and transcatheter aortic valve replacements (TAVR) are well-known treatment options for patients with severe symptomatic aortic stenosis. Based on worldwide registries, approximately 50% of patients who underwent AVR are female patients (2–5).

Although AVR procedures have improved tremendously in recent years, differences in outcomes, including mortality between sexes, remain. Physiological, anatomical, and comorbidity differences between male patients and female patients contribute to the heterogeneity in AVR procedural and long-term outcomes (5–14).

Specifically, female patients tend to be older with a higher frailty score and a lower body mass index (6–13, 15–17). Women have greater periprocedural complications (18–23), such as more frequent bleeding, and more vascular complications than men following the same procedure (8, 14, 18–25). Thus, after AVR, women suffer significantly more than men from in-hospital and 30-day morbidity and mortality (3–10, 13, 21–23). However, although the short-term outcomes are worse, female patients have shown better long-term outcomes with higher survival rates (6, 9, 11, 12, 14, 16, 26–32) known as the sex paradox.

The revolutionary shift from SAVR to a less invasive procedure as TAVR has improved outcomes in women (12, 19, 20, 22–28) as seen in the last decade. Data suggest that short-term outcomes in female patients also improved (21), and the sex-related differences diminished over the years (4, 24, 25, 30–32).

A paucity of literature from the latest TAVR era suggests that the so-called sex paradox may not exist with the new, improved technology and a better patient selection process (4, 9, 30).

This study aimed to investigate temporal trends in sex-related differences in a large cohort of the US database from the National Inpatient Sample (NIS) registry, specifically the in-hospital outcomes in male patients vs. female patients who underwent TAVR procedures during 2011–2017.

## Methods

### Data collection

The data were obtained from the NIS database, the Healthcare Cost and Utilization Project (HCUP), and the Agency for Healthcare Research and Quality (AHRQ) (33). Data from the NIS datasets were de-identified, and therefore, this study was deemed exempt from institutional review by the Human Research Committee.

As described previously (34), the NIS is the largest collection of all-payer data on inpatient hospitalizations in the United States. The dataset represents an approximate 20% stratified sample of all inpatient discharges from US hospitals (35, 36). This information includes patient-level and hospital-level factors such as patient demographic characteristics, primary and secondary diagnoses, procedures, AHRQ comorbidities, length of stay (LOS), hospital region, hospital teaching status, hospital bed size, and cost of hospitalization. National estimates were calculated using the patient-level and hospital-level sampling weights provided by NIS.

For this study, we obtained data for the years 2011 to 2017. The International Classification of Diseases, ICD-9-CM, and ICD-10-CM Revisions were used. Clinical modifications were used for reporting diagnoses and procedures in the NIS database. For each index hospitalization, the database provided a principal discharge diagnosis and a maximum of 14 or 24 additional diagnoses (depending on the year), in addition to a maximum of 15 procedures.

We identified patients aged 18 years or older with a primary diagnosis of aortic stenosis based on ICD-9-CM codes 395.0, 395.2, 396, 396.2, 746.3, 424.1 and based on ICD-10-CM codes I35.0, I35.2, Q23.0, I06.0, I06.2, and I08.0, who underwent in-hospital TAVR procedure codes for PR1-PR15. ICD-9-CM codes 35.05 (trans-femoral) and 35.06 (trans-apical) and ICD-10-CM codes 02RF37Z, 02RF38Z, 02RF3JZ, 02RF3KZ, and X2RF332 (trans-femoral) and 02RF37H, 02RF38H, 02RF3JH, and 02RF3KH (trans-apical) were used.

We used the Deyo-Charlson Comorbidity Index (Deyo-CCI), which predicts the risk of death within 1 year of hospitalization for patients with specific comorbid conditions. Higher Deyo-CCI scores indicated a greater burden of comorbid diseases and were associated with mortality 1 year after admission (37). The Deyo-CCI index has been used extensively in studies from administrative databases, with proven validity in predicting short- and long-term outcomes (38–40). Deyo-CCI uses the ICD-9-CM and ICD-10-CM diagnosis and procedure codes, the administrative data for 17 comorbidities with differential weights of 1 to 6, to calculate the final score index, ranging from 0 to 33. The following patient demographics were collected from the database: age, sex, and race. Prior comorbidities were identified from the AHRQ. Detailed information on Deyo-CCI is provided in Appendix Table 1.

### Outcomes

The primary outcome in this study was all-cause in-hospital mortality. The secondary outcome of interest included in-hospital complications. In-hospital TAVR-related complications were defined (36, 40) as follows: (1) pericardial complications, defined as tamponade, hemopericardium, pericarditis, and pericardiocentesis; (2) cardiac complications (during or resulting from procedure), defined as cardiac block, myocardial infarction, cardiac arrest, congestive heart failure, cardiogenic shock, and others; (3) pulmonary complications, defined as pneumothorax/hemothorax, diaphragm paralysis, postoperative respiratory failure, and other iatrogenic respiratory complications; (4) hemorrhage/hematoma complications, defined as hemorrhage/hematoma complicating a procedure, acute post-hemorrhagic anemia, and hemorrhage requiring transfusion; (5) vascular complications, defined as accidental puncture or laceration during a procedure, injury to blood vessels, arteriovenous fistula, injury to retroperitoneum, vascular complication requiring surgical repair, reopen, and other vascular complications; (6) infection, defined as fever, septicemia, and post-procedural aspiration pneumonia; (7) neurological, defined as nervous system complication, unspecified, central nervous system complication, iatrogenic cerebrovascular infarction or hemorrhage cerebrovascular effect, and transient ischemic attack; (8) diaphragmatic paralysis; (9) acute renal failure; (10) reopen and conversion to open surgery; (11) device-related mechanical complication; (12) paravalvular leak (PVL); and (13) permanent pacemaker implantation (PPM). Detailed information on all ICD-9-CM and ICD-10-CM codes used to identify in-hospital complications is summarized in Appendix Table 2. Length of stay (LOS) was defined as the time interval in days from hospital admission to hospital discharge.

### Statistical analysis

The chi-square (χ^2^) test and Wilcoxon rank sum test were used to compare categorical variables and continuous variables, respectively. Rao-Scott *F*-adjusted chi-square test was used to represent differences in baseline characteristic frequencies of TAVR patients and between-gender differences. LOS (continuous) was compared based on non-parametric confidence intervals according to Zhou and Dinh (41).

### Trends

Piecewise regression analyses were performed to assess temporal trends in TAVR utilization in male patients and in female patients in response to an empirical inflection point corresponding to the early vs. late TAVR eras, before 2014 vs. after 2014. *P*-values were computed using Rao-Scott *F*-adjusted chi-square test to represent differences between year groups in complication frequencies before vs. after 2014.

### Predictors of mortality/complications

We generated a weighted logistic regression model using “TRENDWT” to identify independent predictors of in-hospital complications and in-hospital mortality (further details are found in Appendix Table 3). Congruent with the HCUP NIS design, the hospital identification number was used as a random effect with patient-level factors clustered within hospital-level factors. We retained all predictor variables that were associated with our primary outcome of mortality and secondary outcome of at least one complication with *p* < 0.1 in our final multivariable regression model. For LOS analysis, we generated a logistic regression model. For all analyses, we used SAS^®^ version 9.4 software (SAS Institute Inc., Cary, NC). A *p*-value < 0.05 was considered statistically significant.

## Results

We analyzed data out of 30,153 unweighted hospitalizations in the NIS database from 2011 to 2017. After implementing the weighting method, these represented a total of 150,647 hospitalizations for aortic stenosis in patients who underwent in-hospital TAVR during the index hospitalization.

### Baseline characteristics

In this study, from 2011 to 2017, there were significantly more TAVR procedures performed in men [80,477 (53.4%)] than in women [70,170 (46.6%)]. However, in patients older than 80 years, there was a female predominance (67.7%). Most patients were Caucasian (82.7%), and the mean age was 80.6 ± 8.2 years. Baseline and procedural characteristics categorized by sex are presented in Table 1.

**Table 1:** Baseline and TAVR procedural characteristics categorized by sex.

	**Total**	**Male**	**Female**	***P*-Value**
**Population**, ***n*** **(%)**				
Unweighted	30,153	16,108 (53.4)	14,045 (46.6)	<0.001
Weighted	150,647	80,477 (53.4)	70,170 (46.6)	
**Age, years %**				<0.001
18–49	0.4	0.5	0.3	
50–59	1.6	1.9	1.3	
60–69	8.3	8.7	7.7	
70–79	26.9	28.6	25.0	
>80	62.8	60.3	67.7	
**Race, %**				<0.001
White	82.7	83.8	81.6	
**Comorbidities, %**				
Hypertension	52.3	50.3	54.5	<0.001
Hyperlipidemia	65.6	67.5	63.5	<0.001
Cerebrovascular disease	5.1	5.0	5.2	0.44
Congestive heart failure	28.8	29.3	28.3	0.07
Diabetes mellitus	35.1	37.1	32.8	<0.001
Renal failure	35.9	40.3	30.9	<0.001
Chronic pulmonary disease	30.9	31.0	30.7	0.60
Peripheral vascular disorders	26.8	29.4	23.9	<0.001
Prior CAD/IHD	27.6	30.4	24.3	<0.001
Prior PCI	9.4	10.6	8.1	<0.001
Prior Pacemaker/ICD/CRTD	13.0	15.2	10.5	<0.001
Prior sternotomy, %	32.0	41.3	21.4	<0.001
**Deyo-CCI, %**				<0.001
0	6.3	5.5	7.2	
1	11.2	9.8	12.8	
2 or higher	82.5	84.7	80.0	
**Length of stay (days)**	5.7 ± 0.1	5.5 ± 0.1	6.0 ± 0.1	0.001
**Mortality**	2.3	2.1	2.7	0.001

CAD, cardiovascular disease; CRTD, cardiac resynchronization therapy devices; Deyo-CCI, Deyo-Charlson Comorbidity Index; ICD, implantable cardioverter-defibrillator; IHD, ischemic heart disease; TAVR, transcatheter aortic valve replacement.

Regardless of sex, most patients also presented with hypertension and hyperlipidemia (>50%). Male patients had significantly higher Deyo-CCI score and higher proportions of hyperlipidemia, diabetes mellitus, chronic renal disease, peripheral artery disease, and coronary artery disease and tended to be smokers. Furthermore, male patients had a higher rate of previous cardiac intervention as prior percutaneous coronary intervention and a higher rate of prior device implantation (Table 1).

### AVR utilization trends

**Figure 1:**
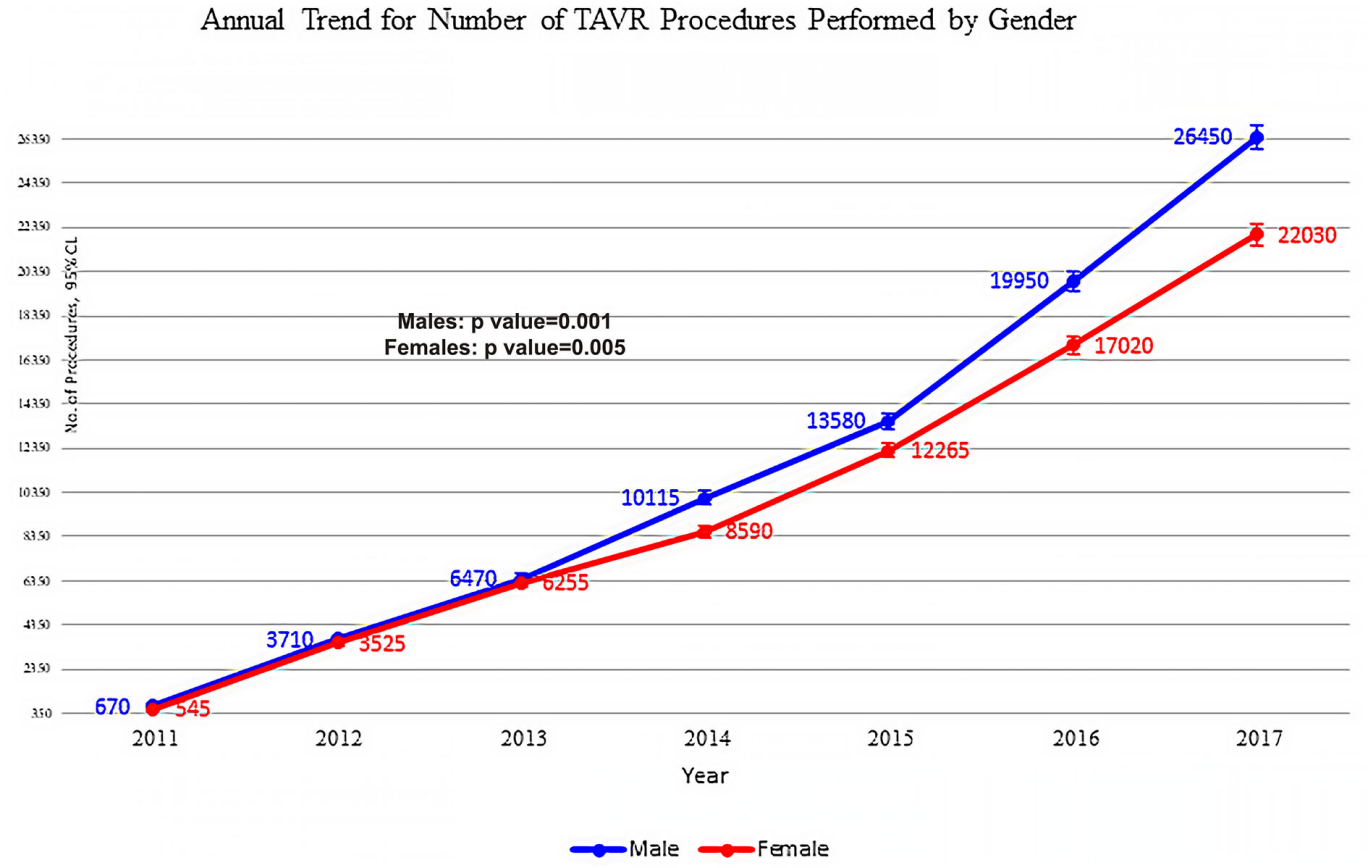
Annual trend for the number of procedures performed by sex and procedure. Piecewise linear regression between years before and after 2014; *p*-value is 0.001 for male patient and 0.005 for female patient.

Our data show that the annual number of TAVR procedures has increased from 1,215 in 2011 to 48,480 in 2017 (Figure 1). The same trend characterized both female patients and male patients. A significant and steady upward trend was observed for TAVR procedures in both sexes, rising from 670 in 2011 to 26,450 in 2017 for male patients and from 545 in 2011 to 22,030 in 2017 for female patients (Figure 1).

Using a piecewise regression analysis, a significant steady upward trend was observed for TAVR procedures from 2011 to 2017, with an additionally pointed elevation after 2014 in both sexes (*p* = 0.001 for male patients and *p* < 0.005 for female patients) (Figure 1).

### Clinical outcomes

**Figure 2:**
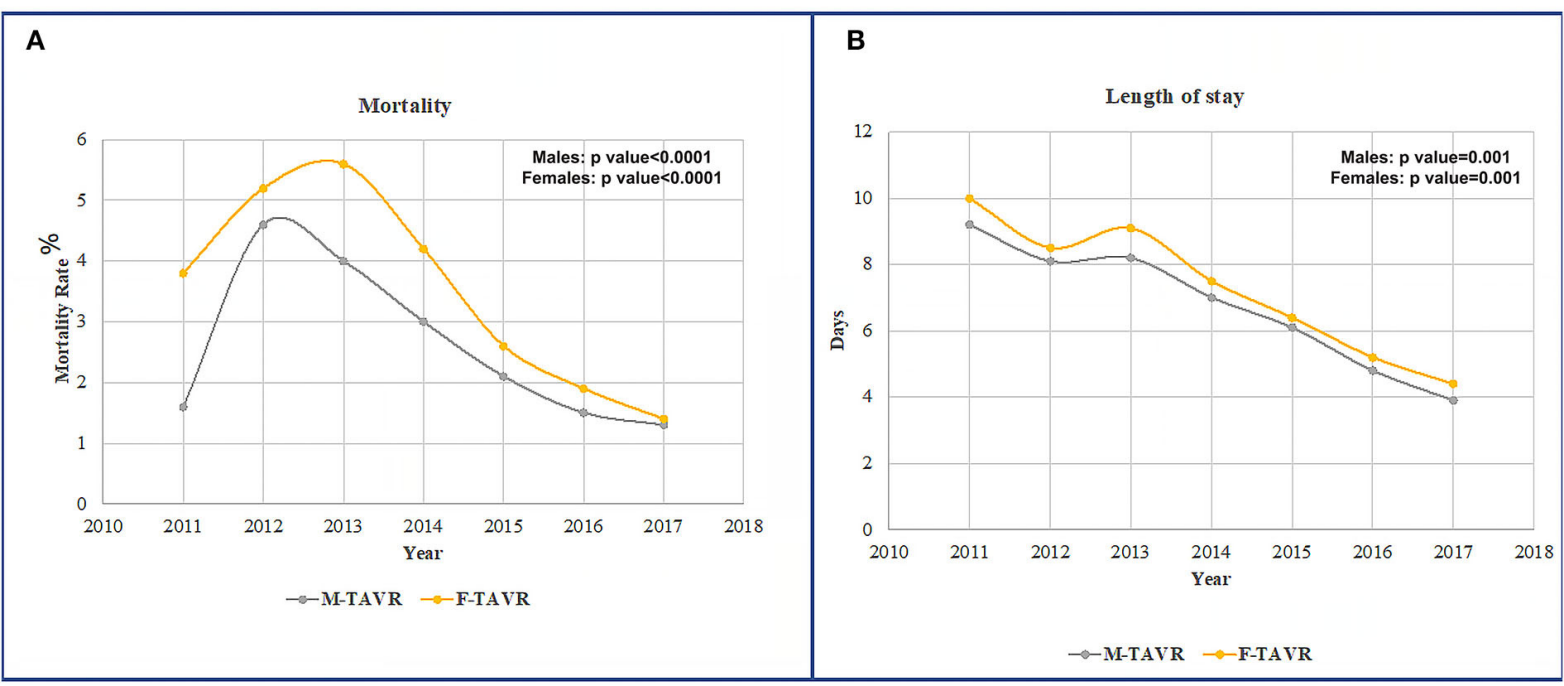
**(A)** Mortality rates in male patients vs. female patients after TAVR during 2011–2017. **(B)** LOS rates in male patients vs. female patients after TAVR during 2011–2017.

All-cause in-hospital mortality during the study period was 2.3% (Table 1). Differences in mortality among sexes were observed, with higher in-hospital mortality in women (2.7%) than in men (2.1%) (Figure 2A). Over time, there was improved mortality, with similar trends observed in both men and women, peaking in 2013 and dropping down to a minimum in 2017, the last observation year (Figure 2A).

Women had a longer LOS than men (6.0 ± 0.1 vs. 5.5 ± 0.1, *p* = 0.0001, respectively). TAVR LOS decreased significantly in men and women from 2011 to 2017 but was still higher in women (Figure 2B).

### Procedural complications

Procedural complications categorized by sex are presented in Table 2. Following TAVR, female patients had significantly higher rates of pericardial, cardiac, pulmonary, hemorrhagic, vascular, and neurological complications (Table 2). Acute renal failure, device-related mechanical complications, and pacemaker implantation were significantly higher in male patients, although similar downward trends were observed for both sexes (Table 2).

**Table 2:** TAVR procedural complications categorized by sex.

	**Total**	**Male**	**Female**	***P*-Value**
Pericardial	2.7	1.8	3.7	<0.001
Cardiac	9.0	8.0	10.1	<0.001
Pulmonary	5.1	4.7	5.6	0.001
Hemorrhage/Hematoma	1.4	1.1	1.8	<0.001
Vascular	4.3	3.7	5.1	<0.001
Infection	2.1	2.1	2.2	0.64
Neurological	0.9	0.8	1.1	0.001
Acute renal failure	12.3	13.3	11.1	<0.001
Cardiogenic shock	2.3	2.4	2.2	0.11
Diaphragmatic paralysis	0.1	0.2	0.1	0.27
Re-open	0.2	0.2	0.3	0.11
Mechanical complication device related	2.3	2.5	1.9	<0.001
Pacemaker	9.9	10.5	9.4	0.001
Paravalvular leak	0.9	1.0	0.8	0.09

**Figure 3:**
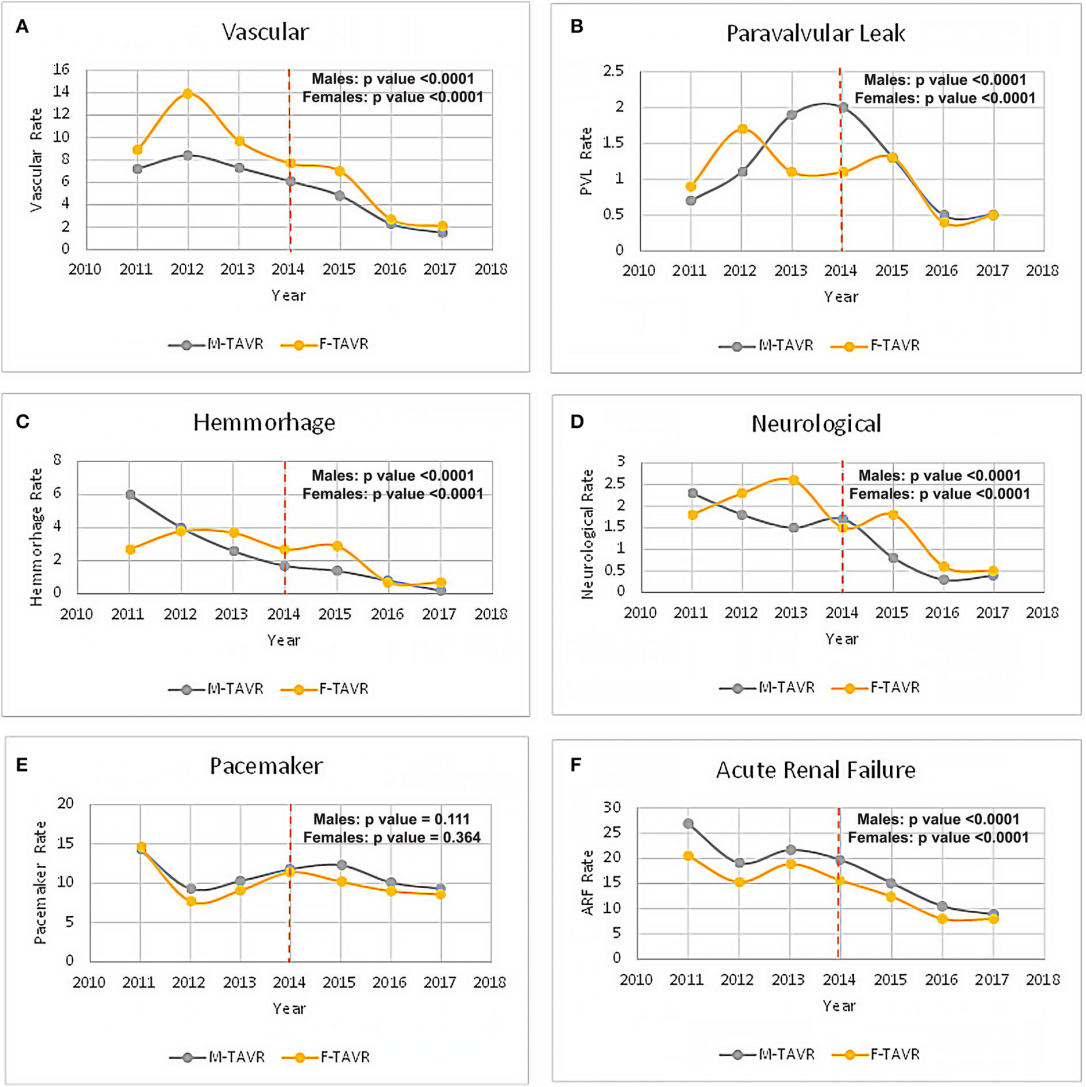
Complications following TAVR in male patients vs. female patients in the early vs. late TAVR era. **(A)** Vascular complication. **(B)** Paravalvular leak. **(C)** Hemorrhage complication. **(D)** Neurological complication. **(E)** New pacemaker implantation. **(F)** Acute renal failure.

Trends in complication rates during 2011–2017 are presented in Figures 3A–F. A significant downward trend was observed in a majority of the complications rate in the early vs. late TAVR era in both male and female patients (Figures 3A–F).

### Multivariable analysis

The multivariable regression model analysis adjusted for potential confounders is presented in Table 3. Women had a higher mortality risk with an odds ratio of 1.26 (95%CI 1.18–1.35), *p* < 0.001 (Table 3).

**Table 3:** Multivariate analysis for predictors of mortality from 2011 to 2017 in the TAVR cohort.

**Predictor**	**Odds ratio (95% CI)**	***P*-Value**
**Gender**		<0.001
Male	1.00 (reference)	N/A
Female	1.26 (1.18, 1.35)	<0.001
**Age group**		<0.001
18–49 yrs	1.00 (reference)	N/A
50–59 yrs	1.90 (0.97, 3.72)	0.060
60–69 yrs	1.27 (0.67, 2.42)	0.460
70–79 yrs	1.34 (0.71, 2.52)	0.362
80–89 yrs	1.51 (0.80, 2.82)	0.202
90 yrs or older	2.11 (1.12, 3.97)	0.020
**Race**		<0.001
White	1.00 (reference)	N/A
Asian or Pacific Islander	0.94 (0.67, 1.32)	0.718
Black	0.59 (0.47, 0.74)	<0.001
Hispanic	1.32 (1.13, 1.54)	<0.001
Native American	2.11 (1.25, 3.57)	0.005
***Comorbidities***		
**Hypertension**		<0.001
No	1.00 (reference)	N/A
Yes	0.49 (0.46, 0.53)	<0.001
**Hyperlipidemia**		<0.001
No	1.00 (reference)	N/A
Yes	0.48 (0.45, 0.51)	<0.001
**Cerebrovascular disease**		0.058
No	1.00 (reference)	N/A
Yes	1.13 (1.00, 1.28)	0.058
**Congestive heart failure**		0.025
No	1.00 (reference)	N/A
Yes	1.10 (1.01, 1.20)	0.025
**Diabetes mellitus**		<0.001
No	1.00 (reference)	N/A
Yes	0.72 (0.66, 0.77)	<0.001
**Renal failure**		<0.001
No	1.00 (reference)	N/A
Yes	1.47 (1.37, 1.57)	<0.001
**Chronic pulmonary disease**		<0.001
No	1.00 (reference)	N/A
Yes	1.17 (1.09, 1.26)	<0.001
**Peripheral vascular disorders**		<0.001
No	1.00 (reference)	N/A
Yes	1.30 (1.21, 1.39)	<0.001
**Prior CAD/IHD**		<0.001
No	1.00 (reference)	N/A
Yes	0.81 (0.74, 0.89)	<0.001
**Prior PCI**		<0.001
No	1.00 (reference)	N/A
Yes	0.59 (0.52, 0.67)	<0.001
**Prior cardiac surgery**		<0.001
No	1.00 (reference)	N/A
Yes	0.61 (0.56, 0.67)	<0.001
**Deyo-CCI**		
0	1.00 (reference)	N/A
1	1.09 (0.91, 1.32)	0.354
2 or higher	1.49 (1.28, 1.74)	<0.001

AVR, aortic valve replacement; SAVR, surgical aortic valve replacement; TAVR, transcatheter aortic valve replacement; IHD, ischemic heart disease; PCI, percutaneous coronary intervention; NA, no available.

Table 4 presents separate analyses for each gender. Women had fewer comorbidities independently associated with mortality than men. Renal failure, peripheral vascular disease, and higher Deyo-CCI score were independent risk factors for both sexes, while female patients with peripheral vascular disease had a higher probability of mortality (Table 4).

**Table 4:** Multivariable analysis for predictors of TAVR mortality from 2011 to 2017 by sex.

	**Male**		**Female**	
**Predictor**	**Odds ratio (95% CI)**	***P*-Value**	**Odds ratio (95% CI)**	***P*-Value**
***Comorbidities***				
**Hypertension**		<0.001		<0.001
No	1.00 (reference)	N/A	1.00 (reference)	N/A
Yes	0.53 (0.47, 0.59)	<0.001	0.46 (0.42, 0.52)	<0.001
**Hyperlipidemia**		<0.001		<0.001
No	1.00 (reference)	N/A	1.00 (reference)	N/A
Yes	0.46 (0.42, 0.51)	<0.001	0.50 (0.45, 0.55)	<0.001
**Cerebrovascular disease**		<0.001		0.311
No	1.00 (reference)	N/A	1.00 (reference)	N/A
Yes	1.40 (1.18, 1.66)	<0.001	0.91 (0.76, 1.09)	0.311
**Congestive heart failure**		<0.001		0.651
No	1.00 (reference)	N/A	1.00 (reference)	N/A
Yes	1.25 (1.11, 1.41)	<0.001	0.97 (0.86, 1.10)	0.651
**Diabetes mellitus**		<0.001		<0.001
No	1.00 (reference)	N/A	1.00 (reference)	N/A
Yes	0.77 (0.69, 0.86)	<0.001	0.66 (0.59, 0.74)	<0.001
**Renal failure**		<0.001		<0.001
No	1.00 (reference)	N/A	1.00 (reference)	N/A
Yes	1.55 (1.40, 1.71)	<0.001	1.40 (1.27, 1.54)	<0.001
**Chronic pulmonary disease**		<0.001		0.224
No	1.00 (reference)	N/A	1.00 (reference)	N/A
Yes	1.30 (1.18, 1.44)	<0.001	1.06 (0.96, 1.18)	0.224
**Peripheral vascular disorders**		<0.001		<0.001
No	1.00 (reference)	N/A	1.00 (reference)	N/A
Yes	1.27 (1.14, 1.40)	<0.001	1.33 (1.20, 1.47)	<0.001
**Prior CAD/IHD**		0.004		0.001
No	1.00 (reference)	N/A	1.00 (reference)	N/A
Yes	0.81 (0.71, 0.94)	0.004	0.82 (0.73, 0.93)	0.001
**Prior sternotomy**		<0.001		<0.001
No	1.00 (reference)	N/A	1.00 (reference)	N/A
Yes	0.61 (0.55, 0.69)	<0.001	0.60 (0.52, 0.69)	<0.001
**Deyo-CCI**		<0.001		0.001
0	1.00 (reference)	N/A	1.00 (reference)	N/A
1	0.97 (0.71, 1.33)	0.852	1.16 (0.92, 1.47)	0.198
2 or higher	1.69 (1.31, 2.17)	<0.001	1.37 (1.13, 1.67)	0.002

Deyo-CCI, Deyo-Charlson Comorbidity Index; IHD, ischemic heart disease; TAVR, transcatheter aortic valve replacement; NA, no available.

## Discussion

This retrospective study found significant differences in male patients vs. female patients undergoing TAVR procedures between 2011 and 2017. Differences in in-hospital mortality rates among sexes were observed for TAVR, with higher in-hospital mortality in women than in men.

After TAVR procedures, this study observed that women had significantly higher in-hospital mortality rates than men over the years. LOS was also significantly higher in women compared to men. There was a peak in mortality and LOS around 2014 and then a steady and significant decrease in these clinical outcomes over the years in both groups. Still, women have poor short-term outcomes compared to men.

The vastly increasing number of procedures performed led to better outcomes and fewer complications over the years due to more experienced operators, better patient selection, and better technology (42, 43). Hence the notion that women will benefit from these advances and have better or equal outcomes than men, unlike their worse outcomes compared to men with SAVR (30, 44).

The “sex paradox” describes the discordance between the higher rates of short-term mortality and complications in women compared to better long-term survival (13, 22, 45–48). Fewer baseline comorbidities could explain this paradox in women who appear to start the process in better general health (13, 23), which might be the explanation for findings in previous papers suggesting lower long-term mortality in women. They are healthier, and thus, if they do not suffer short-term complications, they live longer (4, 29). As reproduced in this study, for various reasons, women suffer more periprocedural complications (23), primarily vascular and bleeding (49). Perhaps this is not a paradox at all but a manifestation of women's higher rates of periprocedural complications. Female patients were found to have smaller anatomy of the atrioventricular area and smaller annular diameters (50). Women also have significantly smaller vascular anatomy, which could be associated with higher rates of vascular complications (51). Data from the TVT registry showed a significantly higher rate of TAVR performed *via* alternative access, 45% in women compared to 35% in men, possibly explaining their higher complication rate (16). Our data show that >93% trans-femoral approach was used in the study cohort, which included the late TAVR era. This could be the explanation for vascular complications reduction in both women and men. Further investigation into this phenomenon is warranted. The increase in the trans-femoral approach in the late TAVR era could be explained by better patient selection and devices and delivery systems that improved significantly and by the learning curve of new technology that entered the market.

The periprocedural complication rate decreased significantly between the early and late periods of this study with 2014 being the cutoff point. This is supported in other studies as well (23, 24, 27, 29). We tried to understand whether this reduction affects the mortality differences between the sex groups. The observation that women are more susceptible to early complications and thus have higher in-hospital mortality persisted throughout the study periods. Despite the decline in mortality in the late study period, the difference remained, with higher mortality in women participants compared to men. More extensive studies focusing on female early mortality in these procedures are crucial for understanding how to improve outcomes in this population.

Our study should be interpreted in the context of several limitations. First, the NIS database is a retrospective administrative database containing discharge-level records and is susceptible to coding errors, and reporting may not be consistent across different institutions. Second, the NIS does not include detailed clinical information and therefore cannot rule out residual confounding of the associations we observed. Additionally, the NIS precluded using follow-up beyond the same index hospitalization. These limitations are counterbalanced by the real-world, nationwide nature of the data, as well as the mitigation of reporting bias introduced by selective publication of results from specialized centers. We used a logistic model for the complications.

In conclusion, while a significant downward trend in complication rates was observed, in-hospital mortality remains higher in female patients. This should be further investigated to understand the mechanism behind this phenomenon to reduce early mortality in this group.

## Data availability statement

The data analyzed in this study is subject to the following licenses/restrictions: The data were obtained from the NIS database, the Healthcare Cost and Utilization Project (HCUP), and the Agency for Healthcare Research and Quality (AHRQ). Data from the NIS datasets were de-identified and therefore this study was deemed exempt from institutional review by the Human Research Committee. Requests to access these datasets should be directed to hcup@ahrq.gov.

## Ethics statement

Ethical review and approval was not required for the study on human participants in accordance with the local legislation and institutional requirements. Written informed consent for participation was not required for this study in accordance with the national legislation and the institutional requirements.

## Author contributions

GE-G and GR conceived the idea and design of the study and drafted the manuscript. ER and MY drafted the manuscript. SC contributed to the data analysis and interpretation and provided revisions to the manuscript. HW and SR contributed to the data interpretation and provided major revisions to the manuscript. DP provided major revisions to the manuscript. OA is the principal investigator, conceived the idea and design of the study, and provided revisions to the manuscript. GE-G had access to all the study data, takes responsibility for the accuracy of the analysis, has the authority over manuscript preparation, and the decision to submit the manuscript for publication. All authors read and approved the manuscript.

## Conflict of interest

The authors declare that the research was conducted in the absence of any commercial or financial relationships that could be construed as a potential conflict of interest.

## Publisher's note

All claims expressed in this article are solely those of the authors and do not necessarily represent those of their affiliated organizations, or those of the publisher, the editors and the reviewers. Any product that may be evaluated in this article, or claim that may be made by its manufacturer, is not guaranteed or endorsed by the publisher.
