# Supplementary material for: Sex difference and outcome trends following transcatheter aortic valve replacement
Source: Front Cardiovasc Med. 2022 Oct 18;9:1013739. doi: 10.3389/fcvm.2022.1013739 (PMC9623151; doi:10.3389/fcvm.2022.1013739)
Supplement: Supplementary file 1 [file Data_Sheet_1.docx]

**Appendix:**

**Appendix 1:** Deyo-Charlson Comorbidity Index (Deyo-CCI score), ICD-9-CM and ICD-10-CM codes

| **ICD-9 CM codes** | **Condition** | **Score** |
| --- | --- | --- |
| 410 – 410.9 | Myocardial infarction | 1 |
| 428 – 428.9 | Congestive heart failure | 1 |
| 433.9, 441 – 441.9, 785.4, V43.4 | Peripheral vascular disease | 1 |
| 430 – 438 | Cerebrovascular disease | 1 |
| 290 – 290.9 | Dementia | 1 |
| 490 – 496, 500 – 505, 506.4 | Chronic pulmonary disease | 1 |
| 710.0, 710.1, 710.4, 714.0 – 714.2, 714.81, 725 | Rheumatologic disease | 1 |
| 531 – 534.9 | Peptic ulcer disease | 1 |
| 571.2, 571.5, 571.6, 571.4 – 571.49 | Mild liver disease | 1 |
| 250 – 250.3, 250.7 | Diabetes | 1 |
| 250.4 – 250.6 | Diabetes with chronic complications | 2 |
| 344.1, 342 – 342.9 | Hemiplegia or paraplegia | 2 |
| 582 – 582.9, 583 – 583.7, 585, 586, 588 – 588.9 | Renal disease | 2 |
| 140-172.9, 174-195.8, 200-208.9 | Any malignancy including leukemia and lymphoma | 2 |
| 572.2 – 572.8 | Moderate or severe liver disease | 3 |
| 196-199.1 | Metastatic solid tumor | 6 |
| 042 – 044.9 | Acquired Immunodeficiency syndrome (AIDS) | 6 |

| **ICD-10 CM codes** | **Condition** | **Score** |
| --- | --- | --- |
| I21.x, I22.x, I25.2 | Myocardial infarction | 1 |
| I09.9, I11.0, I13.0, I13.2, I25.5, I42.0, I42.5-I42.9, I43.x, I50.x, P29.0 | Congestive heart failure | 1 |
| I70.x, I71.x, I73.1, I73.8, I73.9, I77.1, I79.0,  I79.2, K55.1, K55.8, K55.9, Z95.8, Z95.9 | Peripheral vascular disease | 1 |
| G45.x, G46.x, H34.0, I60.x-I69.x | Cerebrovascular disease | 1 |
| F00.x-F03.x, F05.1, G30.x, G31.1 | Dementia | 1 |
| I27.8, I27.9, J40.x-J47.x, J60.x-J67.x, J68.4, J70.1, J70.3 | Chronic pulmonary disease | 1 |
| M05.x, M06.x, M31.5, M32.x-M34.x, M35.1, M35.3, M36.0 | Rheumatologic disease | 1 |
| K25.x-K28.x | Peptic ulcer disease | 1 |
| B18.x, K70.0-K70.3, K70.9, K71.3-K71.5, K71.7, K73.x, K74.x, K76.0, K76.2-K76.4, K76.8, K76.9, Z94.4 | Mild liver disease | 1 |
| E10.0, E10.l, E10.6, E10.8, E10.9, E11.0, E11.1, E11.6, E11.8, E11.9, E12.0, E12.1, E12.6, E12.8, E12.9, E13.0, E13.1, E13.6, E13.8, E13.9, E14.0, E14.1, E14.6, E14.8, E14.9 | Diabetes | 1 |
| E10.2-E10.5, E10.7, E11.2-E11.5, E11.7, E12.2-E12.5, E12.7, E13.2-E13.5, E13.7, E14.2-E14.5, E14.7 | Diabetes with chronic complications | 2 |
| G04.1, G11.4, G80.1, G80.2, G81.x, G82.x, G83.0-G83.4, G83.9 | Hemiplegia or paraplegia | 2 |
| I12.0, I13.1, N03.2-N03.7, N05.2-N05.7, N18.x, N19.x, N25.0, Z49.0-Z49.2, Z94.0, Z99.2 | Renal disease | 2 |
| C00.x-C26.x, C30.x-C34.x, C37.x-C41.x, C43.x, C45.x-C58.x, C60.x-C76.x, C81.x-C85.x, C88.x, C90.x-C97.x | Any malignancy including leukemia and lymphoma | 2 |
| I85.0, I85.9, I86.4, I98.2, K70.4, K71.1, K72.1, K72.9, K76.5, K76.6, K76.7 | Moderate or severe liver disease | 3 |
| C77.x-C80.x | Metastatic solid tumor | 6 |
| B20.x-B22.x, B24.x | Acquired Immunodeficiency syndrome (AIDS) | 6 |

**Appendix 2:** In-hospital complication codes for ICD-9-CM and ICD-10-CM

| **Complication** | **ICD-9-CM Code(s)** | **ICD-10-CM Code(s)** |
| --- | --- | --- |
| **Pericardial** |  |  |
| Hemopericardium | 423.0 | I31.2 |
| Tamponade | 423.3 | [I31.4](https://www.icd10data.com/ICD10CM/Codes/I00-I99/I30-I52/I31-/I31.4) |
| Pericardiocentesis | 37.0 | OW9DXXX, OW9CXXX, 0W9D40Z |
| Acute pericarditis | 420.9 | I30.1, I30.8, I30.9 |
| **Cardiac**  (including post-operative cardiac block, myocardial infarction,  cardiac arrest, and congestive heart failure) | 997.1 | I97.710, I97.110, I97.120, I97.130, I97.190, I97.88, I97.89, I97.711, I97.790  I97.XXX |
| **Pulmonary** |  |  |
| Pneumothorax/hemothorax | 512.1-512.2, 511.8 | [J95.811](https://www.icd10data.com/ICD10CM/Codes/J00-J99/J95-J95/J95-/J95.811), [J95.812](https://www.icd10data.com/ICD10CM/Codes/J00-J99/J95-J95/J95-/J95.812), J95.830, J95.831, J94.2 |
| Diaphragm paralysis | 519.4 | [J98.6](https://www.icd10data.com/ICD10CM/Codes/J00-J99/J96-J99/J98-/J98.6) |
| Post-operative Respiratory Failure | 518.51, 518.53 | [J95.821](https://www.icd10data.com/ICD10CM/Codes/J00-J99/J95-J95/J95-/J95.821), [J96.00](https://www.icd10data.com/ICD10CM/Codes/J00-J99/J96-J99/J96-/J96.00), [J95.822](https://www.icd10data.com/ICD10CM/Codes/J00-J99/J95-J95/J95-/J95.822), [J96.20](https://www.icd10data.com/ICD10CM/Codes/J00-J99/J96-J99/J96-/J96.20) |
| Other iatrogenic Respiratory Complications | 997.3 | J95.88, J95.89, J95.851, J95.859 |
| **Hemorrhage/Hematoma** |  |  |
| Hemorrhage/hematoma complicating a procedure | 998.11-998.12 | [I97.411](https://www.icd10data.com/ICD10CM/Codes/I00-I99/I95-I99/I97-/I97.411), [I97.418](https://www.icd10data.com/ICD10CM/Codes/I00-I99/I95-I99/I97-/I97.418), [I97.42](https://www.icd10data.com/ICD10CM/Codes/I00-I99/I95-I99/I97-/I97.42), [I97.611](https://www.icd10data.com/ICD10CM/Codes/I00-I99/I95-I99/I97-/I97.611)  [I97.618](https://www.icd10data.com/ICD10CM/Codes/I00-I99/I95-I99/I97-/I97.618), [I97.620](https://www.icd10data.com/ICD10CM/Codes/I00-I99/I95-I99/I97-/I97.620), [I97.411](https://www.icd10data.com/ICD10CM/Codes/I00-I99/I95-I99/I97-/I97.411), [I97.418](https://www.icd10data.com/ICD10CM/Codes/I00-I99/I95-I99/I97-/I97.418), [I97.42](https://www.icd10data.com/ICD10CM/Codes/I00-I99/I95-I99/I97-/I97.42), [I97.621](https://www.icd10data.com/ICD10CM/Codes/I00-I99/I95-I99/I97-/I97.621), [I97.631](https://www.icd10data.com/ICD10CM/Codes/I00-I99/I95-I99/I97-/I97.631), [I97.638](https://www.icd10data.com/ICD10CM/Codes/I00-I99/I95-I99/I97-/I97.638) |
| Acute post-hemorrhagic anemia | 285.1 | D62 |
| Hemorrhage requiring transfusion | (998.11-998.12, 285.1) AND (99.00-99.09) | [I97.411](https://www.icd10data.com/ICD10CM/Codes/I00-I99/I95-I99/I97-/I97.411), [I97.418](https://www.icd10data.com/ICD10CM/Codes/I00-I99/I95-I99/I97-/I97.418), [I97.42](https://www.icd10data.com/ICD10CM/Codes/I00-I99/I95-I99/I97-/I97.42), [I97.611](https://www.icd10data.com/ICD10CM/Codes/I00-I99/I95-I99/I97-/I97.611)  [I97.618](https://www.icd10data.com/ICD10CM/Codes/I00-I99/I95-I99/I97-/I97.618), [I97.620](https://www.icd10data.com/ICD10CM/Codes/I00-I99/I95-I99/I97-/I97.620), [I97.411](https://www.icd10data.com/ICD10CM/Codes/I00-I99/I95-I99/I97-/I97.411), [I97.418](https://www.icd10data.com/ICD10CM/Codes/I00-I99/I95-I99/I97-/I97.418), [I97.42](https://www.icd10data.com/ICD10CM/Codes/I00-I99/I95-I99/I97-/I97.42), [I97.621](https://www.icd10data.com/ICD10CM/Codes/I00-I99/I95-I99/I97-/I97.621), [I97.631](https://www.icd10data.com/ICD10CM/Codes/I00-I99/I95-I99/I97-/I97.631), [I97.638](https://www.icd10data.com/ICD10CM/Codes/I00-I99/I95-I99/I97-/I97.638)  AND 3023XXX, 3024XXX  30233H0, 30233N0, 30243H0, 30243N0, 30233H1, 30243H1, 30233W0, 30243W0, 30233N1, 30233P1, 30243P1 |
| **Vascular** |  |  |
| Accidental puncture or laceration during a procedure | 998.2, e8700-e709 | [I97.51](https://www.icd10data.com/ICD10CM/Codes/I00-I99/I95-I99/I97-/I97.51), [I97.52](https://www.icd10data.com/ICD10CM/Codes/I00-I99/I95-I99/I97-/I97.52) |
| Injury to blood vessels | 900-904 | S25.X, S35.X |
| Arteriovenous Fistula | 447.0 | I77.0 |
| Injury to retroperitoneum | 868.04 | [S36.899A](https://www.icd10data.com/ICD10CM/Codes/S00-T88/S30-S39/S36-/S36.899A) |
| Vascular complication requiring surgical/Percutaneous repair | 39.30, 39.31, 39.32, 39.41, 39.49, 39.52, 39.53, 39.56, 39.59, 39.90, 39.91, 39.98, 39.99 | [03QY0ZZ](https://www.icd10data.com/ICD10PCS/Codes/0/3/Q/Y/03QY0ZZ), [03QY3ZZ](https://www.icd10data.com/ICD10PCS/Codes/0/3/Q/Y/03QY3ZZ), [03QY4ZZ](https://www.icd10data.com/ICD10PCS/Codes/0/3/Q/Y/03QY4ZZ)  [04QY0ZZ](https://www.icd10data.com/ICD10PCS/Codes/0/4/Q/Y/04QY0ZZ), [04QY3ZZ](https://www.icd10data.com/ICD10PCS/Codes/0/4/Q/Y/04QY3ZZ), [04QY4ZZ](https://www.icd10data.com/ICD10PCS/Codes/0/4/Q/Y/04QY4ZZ)  [05QY0ZZ](https://www.icd10data.com/ICD10PCS/Codes/0/5/Q/Y/05QY0ZZ), [05QY3ZZ](https://www.icd10data.com/ICD10PCS/Codes/0/5/Q/Y/05QY3ZZ), [05QY4ZZ](https://www.icd10data.com/ICD10PCS/Codes/0/5/Q/Y/05QY4ZZ)  [06QY0ZZ](https://www.icd10data.com/ICD10PCS/Codes/0/6/Q/Y/06QY0ZZ), [06QY3ZZ](https://www.icd10data.com/ICD10PCS/Codes/0/6/Q/Y/06QY3ZZ), [06QY4ZZ](https://www.icd10data.com/ICD10PCS/Codes/0/6/Q/Y/06QY4ZZ)  [02QW0ZZ](https://www.icd10data.com/ICD10PCS/Codes/0/2/Q/W/02QW0ZZ), [02QW3ZZ](https://www.icd10data.com/ICD10PCS/Codes/0/2/Q/W/02QW3ZZ), [02QX4ZZ](https://www.icd10data.com/ICD10PCS/Codes/0/2/Q/X/02QX4ZZ)  [03Q00ZZ](https://www.icd10data.com/ICD10PCS/Codes/0/3/Q/0/03Q00ZZ), [03Q03ZZ](https://www.icd10data.com/ICD10PCS/Codes/0/3/Q/0/03Q03ZZ), 03Q04ZZ,  03Q10ZZ, 03Q13ZZ, 03Q14ZZ, 03Q20ZZ, 03Q23ZZ, 03Q24ZZ, 03Q30ZZ, 03Q40ZZ, 03Q33ZZ, 03Q43ZZ, 03Q44ZZ, 03Q50ZZ, 03Q53ZZ, 03Q54ZZ, 03Q60ZZ, 03Q63ZZ, 03Q64ZZ, 03Q74ZZ 03Q70ZZ, 03Q73ZZ, 03Q80ZZ, 03Q83ZZ, 03Q84ZZ, 03Q90ZZ, 03Q93ZZ, 03Q94ZZ, 03QA0ZZ, 03QA3ZZ, 03QA4ZZ, 03QB0ZZ, 03QB3ZZ, 03QB4ZZ,03QC0ZZ, 03QC3ZZ, 03QC4ZZ,03QY0ZZ, 03QY3ZZ,03QY4ZZ,04Q00ZZ,  04Q03ZZ, 04QC0ZZ, 04QC3ZZ  04Q04ZZ, 04QC4ZZ,04QD0ZZ, 04QD3ZZ,04QD4ZZ, 04QE0ZZ 04QE3ZZ, 04QE4ZZ, 04QF0ZZ, 04QF3ZZ, 04QF4ZZ, 04QH0ZZ, 04QH3ZZ, 04QH4ZZ,04QJ0ZZ  04QJ3ZZ, 04QJ4ZZ,04QK0ZZ, 04QK3ZZ, 04QL0ZZ, 04QL3ZZ, 04QL4ZZ, 04QY0ZZ, 04QY3ZZ |
| Other vascular complications | 997.2, 997.7 | [T81.719A](https://www.icd10data.com/ICD10CM/Codes/S00-T88/T80-T88/T81-/T81.719A) , [T81.72XA](https://www.icd10data.com/ICD10CM/Codes/S00-T88/T80-T88/T81-/T81.72XA)  T82.837, T82.838 |
| **Infection** |  |  |
| Fever | 780.60, 780.62 | T82.6, T82.7, R50.82 |
| Septicemia | 038.*, 995.91-995.92, 998.02, 790.7 | A41.9, A65.20, [T81.12XA](https://icd.codes/icd10cm/T8112XA) |
| Post-procedural aspiration pneumonia | 997.32 | J95.89 |
| **Neurological** |  |  |
| Nervous system complication, unspecified | 997.00 | [G97.81](https://www.icd10data.com/ICD10CM/Codes/G00-G99/G89-G99/G97-/G97.81) |
| Central nervous system complication | 997.01 | G97.81, G97.82 |
| Iatrogenic cerebrovascular infarction or hemorrhage | 997.02 | [I97.811](https://www.icd10data.com/ICD10CM/Codes/I00-I99/I95-I99/I97-/I97.811), [I97.821](https://www.icd10data.com/ICD10CM/Codes/I00-I99/I95-I99/I97-/I97.821)  I97.810 |
| Transient ischemic attack | 435.9 | [G45.9](https://www.icd10data.com/ICD10CM/Codes/G00-G99/G40-G47/G45-/G45.9), [I67.848](https://www.icd10data.com/ICD10CM/Codes/I00-I99/I60-I69/I67-/I67.848) |
| Any stroke | 430, 431, 432, 433.01, 433.11, 433.21, | 160.9,161.9, 163.22, 163.139, 163.239  163.019,163.119, 163.219 |
| **Acute renal failure** | 584.5-584.9 | N17, N17.1, N17.2, N17.8, N17.9, N99.0, |
| **Cardiogenic shock** | 785.51 | [R57.0](https://www.icd10data.com/ICD10CM/Codes/R00-R99/R50-R69/R57-/R57.0) |
| **Diaphragmatic Paralysis** | 519.4 | [J98.6](https://www.icd10data.com/ICD10CM/Codes/J00-J99/J96-J99/J98-/J98.6) |
| **Re-open:** | 34.03, 39.41 | 0W39OZZ, OW3BOZZ, OW3COZZ, OW3DOZZ, OW3QOZZ |
| **Mechanical complication device related** |  | T82.01, T82.02, T82.03, T82.09  T82,221, T82.222, T82.223, T82.228  T82.817, T82.818 |
| **Paravalvular leak** | [996.02](http://www.icd9data.com/2015/Volume1/800-999/996-999/996/996.02.htm?__hstc=93424706.ea137040e43c48c3aaabd4b8d8fc49ab.1570976617663.1586967485270.1587292359526.33&__hssc=93424706.6.1587292359526&__hsfp=2094543708) | [T82.03XA](https://www.icd10data.com/ICD10CM/Codes/S00-T88/T80-T88/T82-/T82.03XA) |
| **Pacemaker implantation** | 37.80-37.83, 37.94, 37.95, 37.96 | OJH606Z, OJH636Z, 0JH806Z, OJH836Z, OJH60PZ, OJH63PZ, 0JH80PZ, OJH83PZ, OJH604Z, OJH634Z, OJH804Z, OJH834Z, OJH605Z, 0JH635Z, OJH805Z, 0JH835Z, 02H73KZ, O2HK3KZ,02HL3KZ,02HN0KZ, 02HN4KZ, OJH608Z, OJH638Z, OJH808Z, 0JH838Z, 02H60KZ, 02H63KZ, 02H64KZ, 02H70KZ, 02H73KZ, 02H74KZ, 02HK0KZ, 02HK3KZ, 02HK4KZ,02HL0KZ, 02HL3KZ, 02HL4KZ, 0JH608Z, 0JH638Z, 0JH808Z, 0JH838Z, 02H60KZ, 02H63KZ, 02H64KZ, 02H70KZ, 02H73KZ, 02H74KZ, 02HK0KZ, 02HK3KZ, 02HK4KZ, 02HL0KZ, 02HL3KZ, 02HL4KZ, 0JH608Z, 0JH638Z,  0JH808Z, 0JH838Z |

**Appendix 3:**

| Supplemental methods | The NIS provided discharge sample weights that were calculated within each sampling stratum as the ratio of discharges in the universe to discharges in the sample^42^. Prior to 2012, a 20 percent sample from all hospitals in the US providing long-term acute care and 100 percent discharge data from these hospitals were retained. Beginning in 2012, however, the NIS was redesigned to construct reciprocal information, partially 20 percent of discharge records from all hospitals in the sampling frame. These design changes, however, do not limit multi-year analysis. To account for these revisions while performing trend analysis, AHRQ developed new patient-level discharge trend weights for the years prior to 2012. The new trend weights (called “TRENDWT") were intended to be used instead of the earlier NIS weights (called “DISCWT") in years prior to 2012, while performing a multi-year analysis spanning year 2012. Utilizing the new weights resulted in improved national estimates, in addition to allowing for multi-year analysis of trends. |
| --- | --- |
